# Revisiting a hypothesis: the neurovascular unit as a link between major depression and neurodegenerative disorders

**DOI:** 10.3389/fncel.2024.1455606

**Published:** 2024-08-02

**Authors:** Ravi Philip Rajkumar

**Affiliations:** Department of Psychiatry, Jawaharlal Institute of Postgraduate Medical Education, Pondicherry, India

**Keywords:** depression, neurodegenerative disorders, dementia, neurovascular unit, blood-brain barrier, claudin-5

## The neurovascular unit in health and disease

Cerebral homoeostasis requires precise regulation of global and regional blood flow, ensuring an adequate supply of oxygen and nutrients. The physiological process through which this is ensured is known as autoregulation. At the local level, this involves a mechanism through which increased neuronal demand is “coupled” to increased microvascular blood flow (Muoio et al., 2014). The anatomical and physiological substrate of this process is known as the neurovascular unit (NVU). The NVU is a complex of neurons, astrocytes, endothelial cells, smooth muscle cells, and extracellular matrix components that exist in an intimate structural and functional relationship. Neurovascular coupling involves an intricate process of multi-directional communication between these cells. This occurs through gap junctions, adhesion molecules, ion channels, and specific neurotransmitters or neuromodulators such as glutamate, gamma-amino butyric acid (GABA), prostaglandin E_2_, nitric oxide (NO), and neuropeptide Y (NPY). Apart from the fine regulation of cerebral blood flow, the components of the NVU are actively involved in the removal of neurotoxic substances and in the maintenance of the blood-brain barrier (BBB) (Iadecola, 2017; Yao et al., 2023). Over the past two decades, it has been recognized that the NVU plays a central role in the pathophysiology of several neurological disorders, including cerebrovascular disease, multiple sclerosis, cerebral tumors, and neurodegenerative disorders such as Alzheimer's disease (Hawkins and Davis, 2005; McConnell et al., 2017).

## The neurovascular unit and mental disorders

There is evidence from both animal and human research that the NVU is involved in the modulation of both cognitive functions, such as attention and memory, and emotional responses (Dion-Albert et al., 2023). Responding to stressful situations requires changes in both cognition and affect, which result from altered functioning of several brain regions and networks. This process requires the precise regulation of regional cerebral blood flow at the level of the NVU (Han et al., 2020; Alyan et al., 2021). The impact of stress on the NVU, and hence on cognitive and affective functioning, depends on the duration and severity of the stressor. Acute stressors, of the sort commonly encountered in a workplace setting, produce immediate, short-lasting changes in regional cerebral blood flow (Elbau et al., 2018). Chronic or traumatic stress can lead to more long-lasting changes in neurovascular coupling in specific brain circuits, both at rest and during specific tasks (Portes et al., 2019; Park et al., 2021). In translational models, these types of stress have also been found to disrupt the integrity of the BBB, increase the penetration of immune cells into the brain, and alter neural activity related to specific transmitters, such as glutamate. Over time, these changes may even lead to neuronal loss (Alshammari et al., 2020; Welcome and Mastorakis, 2020; Cathomas et al., 2022; Peng et al., 2022). For this reason, it has been suggested that altered NVU functioning may be implicated in stress-related mental disorders, particularly those of a chronic or recurrent nature. These conditions include depression, anxiety disorders, and post-traumatic stress disorder (Dion-Albert et al., 2022; Ni et al., 2022; Yao et al., 2023).

## The neurovascular unit and depression: a hypothesis

The link between the NVU and depression is a particularly intriguing one. Depression, also referred to as major depressive disorder (MDD), is the most common mental disorder worldwide, affecting about 4–5% of the global population (World Health Organization, 2017). Stressful life events, particularly those of a chronic or traumatic nature, are closely related to the onset, course and outcome of MDD (Monroe and Harkness, 2005; Corruble et al., 2006; Strauss et al., 2018; Buckman et al., 2022). In older adults, MDD has often been linked to medical conditions, such as diabetes mellitus, atherosclerosis, or hypertension, that affect the integrity of the NVU, even in the absence of an overt stroke. This has led some authors to propose a “vascular depression” subtype of MDD that encompasses such cases (Jellinger, 2021). It is also recognized that MDD, even in the absence of vascular risk factors, can predispose adults both to cerebrovascular disease and to certain neurodegenerative disorders, such as Parkinson's disease (PD) and Alzheimer's disease (AD) (Wang et al., 2018; Zhao et al., 2022). Treatment with conventional antidepressants is effective only in about 40–60% of patients with MDD, and does not seem to reduce the risk of these complications (Moraros et al., 2017).

In the light of this evidence, Najjar et al. (2013) proposed a hypothesis linking MDD to dysfunction of the NVU. Broadly speaking, this hypothesis postulates that oxidative stress and inflammation, which are known to be associated with MDD, lead to NVU and BBB dysfunction through alterations in endothelial NO levels and endothelial nitric oxide synthase (NOS) “uncoupling”—a process in which NOS uses O_2_ as a substrate instead of L-arginine. This leads to reduced NO—which impairs the normal functioning of the NVU—and the generation of reactive oxygen species, creating a “vicious circle”: inflammation and oxidative stress → NVU dysfunction and BBB hyperpermeability → further neuroinflammation and oxidative stress. These changes could lead to neuronal dysfunction and symptoms of depression. This model has three merits:

- it accounts for the limitations of conventional antidepressants that act mainly through monoamine neurotransmitters,
- it is supported both by animal models of depression, and by clinical evidence suggesting that in some cases, anti-inflammatory or antioxidant treatment can improve depression,
- it provides a cogent mechanistic link between MDD and disorders characterized by more extensive neurovascular dysfunction and inflammatory and oxidative damage to neurons.

In the decade since this hypothesis was proposed, evidence from animal and human research has provided further support for it, and has also elucidated some of the precise molecular mechanisms involved. The aim of this paper is to provide a brief overview of this research, to critically re-evaluate this hypothesis in the light of new data, and to propose two extensions to it. For this purpose, relevant literature over the period 2013–2024 was retrieved from the PubMed, Scopus and ScienceDirect databases using the search terms “depression,” “major depressive disorder,” “major depression,” and “depressive disorder” in conjunction with “neurovascular unit” or “blood-brain barrier”.

## Animal models of neurovascular unit dysfunction in depression

### Alterations in claudin-5 expression

Behaviors similar to those seen in depression can be induced by exposure to chronic experimental stress in mice and rats. Studies of these “rodent models” of depression have implicated claudin-5 in the relationship between stress, NVU dysfunction, and depressive-like behaviors. Claudin-5 is a tight junction protein highly expressed by endothelial cells which plays a vital role in NVU integrity and BBB permeability (Greene et al., 2019). It has been noted that chronic stress is associated with methylation of the claudin-5 gene (*CLDN5*) promoter region in mice, which leads to reduced claudin-5 expression in brain regions such as the prefrontal cortex. In contrast, mice which were resilient to stress showed increased acetylation of the *CLDN5* promoter, leading to increased expression of this protein. Endothelial expression of the transcription factor FoxO1 was found to inhibit *CLDN5* expression and increase vulnerability to the depressive phenotype on exposure to chronic stress. Reduced claudin-5 expression was associated with abnormal NVU vascular morphology in the nucleus accumbens, and with increased permeability of the pro-inflammatory cytokines tumor necrosis factor alpha (TNF-α) and interleukin-6 (IL-6) (Menard et al., 2017; Dudek et al., 2020; Dion-Albert et al., 2022; Sun et al., 2024). Disruption of *CLDN5* using a viral vector was also associated with increased vulnerability to depressive-like behavior in female mice (Dion-Albert et al., 2022). In contrast, treatment with lithium was associated with upregulation of claudin-5 in rats exposed to chronic stress (Taler et al., 2021).

### Immune-inflammatory mechanisms

Apart from claudin-5, changes in the expressions of specific microRNAs have also been noted in relation to NVU changes in rats exposed to stress. Those who were “resilient” and did not develop depression-like behaviors showed increased hippocampal levels of miR-455-3p, while those who were “vulnerable” had increased levels of miR-30e-3p; these changes were correlated with BBB permeability and number of microglia in the ventral hippocampus. Vulnerability was exacerbated by the administration of vascular endothelial growth factor (VEGF)-164, which has pro-inflammatory effects, and inhibited by the administration of the cyclooxygenase-II inhibitor meloxicam (Pearson-Leary et al., 2017). Chronic stress was also associated with increased numbers of neural/glial antigen 2-positive (NG2+) pericytes in the rat hippocampal NVU. A similar increase in NG2+ pericytes was seen after administration of the pro-inflammatory cytokine interleukin-1 beta (IL-1β). These changes could lead to increased BBB permeability (Treccani et al., 2021)

### Animal models of depression comorbid with diabetes mellitus

There is additional evidence implicating NVU dysfunction in animal models of depression occurring in experimentally-induced diabetes mellitus. In these rodents, there was evidence of glutamatergic dysfunction in the hippocampal NVU, with increases in glucocorticoid receptor density and levels of glutamate and the metabotropic glutamate receptor mGluR_2/3_. These changes were associated with alterations in the phosphoinositide 3-kinase (PI3K) pathway, increased neuronal apoptosis, reduced levels of serotonin, and reduced expression of brain-derived neurotrophic factor (BDNF) and the glutamate transporter GLUT-4 (Liu et al., 2018, 2021; Gliozzi et al., 2024).

### Linking stress, depression, and cerebrovascular disease

When considering NVU dysfunction in depression, a question that naturally arises is the role of the NVU in patients with depression and associated cerebrovascular disease, including “vascular depression” and vascular cognitive impairment. Two recent studies have found that mice exposed to chronic stress, which produces depressive-like behaviors, exhibit NVU endothelial dysfunction that is associated with BBB “breakdown”, immune-inflammatory activation, and focal microscopic hemorrhages (Lehmann et al., 2020; Samuels et al., 2023). A certain degree of improvement, characterized by repair of damaged small vessels, was observed when mice were no longer exposed to stress. These findings suggest that the NVU changes associated with stress-induced MDD, if prolonged, can be severe enough to cause local ischemic and hemorrhagic brain damage. Overall, these findings paint a picture of a complex relationship between depression—particularly if arising in the context of stress—and NVU dysfunction that, in some cases, can be severe enough to cause ischemic or inflammatory neuronal injury and predispose to cerebrovascular or neurodegenerative disorders. These changes may be wholly or partially reversible either pharmacologically or through changes in the social environment.

## Research on neurovascular unit dysfunction and depression in humans

### Alterations in claudin-5 expression

Preliminary results in humans also suggest that claudin-5 is involved in the pathogenesis of depression. Studies of postmortem brain tissue from patients with depression has found reduced expression of claudin-5, particularly in the nucleus accumbens and hippocampus, along with increased expression of FoxO1 and of histone deactylase 1 (HDAC1), both of which repress the *CLDN5* gene (Menard et al., 2017; Dudek et al., 2020; Greene et al., 2020). On the other hand, peripheral levels of claudin-5 were increased in a small sample (*n* = 40) of patients with depression compared to healthy controls; in these patients, levels of claudin-5 and TNF-α were positively correlated (Hochman et al., 2023). A study of biobank samples found that functional polymorphisms of the *CLDN5* and *IL6* genes had an interactive effect on vulnerability to depression following exposure to stress (Gal et al., 2023).

### Other putative markers of neurovascular unit dysfunction

E-selectin is an adhesion molecule expressed by neurovascular endothelial cells. Animals exposed to chronic stress show increased E-selectin mRNA expression, which appears to be correlated with increased immune cell recruitment and local inflammation (Sawicki et al., 2015). Women with MDD had elevated serum E-selectin when compared with controls (Dion-Albert et al., 2022) and patients with depression had lower plasma levels of soluble VEGF-165 (Wallensten et al., 2021). Early response to the antidepressant duloxetine was associated with elevated plasma VEGF in patients with MDD (Fornaro et al., 2013). Analysis of postmortem brain tissue from patients with MDD who completed suicide found evidence of lower expression of serpin peptidase inhibitor, clade H (SERPINH1), an inhibitor of proteolytic activity, which may be involved in maintaining endothelial cell-extracellular matrix interactions in the NVU (Pantazatos et al., 2017).

### Neuroimaging

Brain imaging in patients with depression has found evidence of increased perivascular spaces in association with exposure to traumatic stress (Ranti et al., 2022) and increased mean volume transfer constant, a measure of BBB permeability, in the olfactory cortex, caudate nucleus, and thalamus of patients when compared to controls (Shang et al., 2024). In the latter study, BBB permeability in the thalamus and hippocampus was positively correlated with the severity of depressive symptoms.

### Serotonin and glutamate as links between depression and neurovascular unit dysfunction

Serotonin (5-hydroxytryptamine; 5-HT) is a neurotransmitter involved in the regulation of mood, cognition, stress responses, and biological functions such as sleep and appetite (Berger et al., 2009). Despite much debate about their exact significance, alterations in serotonergic transmission have been frequently demonstrated in patients with depression (Moller, 2023), and most of the commonly used antidepressants act as serotonin reuptake inhibitors or receptor modulators (Lochmann and Richardson, 2019). A recent study suggests that serotonin may enhance the expression of claudin-5 by brain endothelial cells through its actions on serotonin type 1A (5HT_1A_) receptors (Sugimoto et al., 2021). Serotonergic antidepressants have been shown to improve endothelial function, as measured using flow-mediated dilation, in patients with MDD (Delialis et al., 2022). Neuronal serotonin levels were significantly reduced in an animal model of diabetes-related depression with associated NVU dysfunction (Liu et al., 2018), and the administration of the antidepressant fluoxetine appeared to “rescue” NVU/BBB changes in an animal model of depression (Sun et al., 2024).

Glutamate is the chief excitatory neurotransmitter in the central nervous system. There is evidence that altered glutamatergic transmission is also an important mechanism linking NVU dysfunction and MDD. Stress is known to alter the release, synaptic effects and clearance of glutamate, and some researchers have proposed a “glutamate hypothesis” of depression akin to the “serotonin hypothesis” (Sanacora et al., 2012). Glutamate receptors and transporters are highly expressed in the NVU, and may play a role in local vasodilation (Parfenova et al., 2012; Morgun et al., 2015). However, at higher levels, glutamate may be toxic to both neurons and the NVU (Castillo et al., 2016). Animal models of comorbid depression and diabetes have shown evidence of elevated glutamate, glutamatergic synaptic dysfunction, and upregulation of glutamate transporters and metabotropic glutamate receptors (Liu et al., 2021; Gliozzi et al., 2024). It is possible that in milder forms of depression, stress-induced changes in glutamate levels may affect the regulation of cerebral blood flow by the NVU in key brain regions. In chronic MDD, persistent elevations in glutamate may lead to neuronal death and NVU remodeling. Therefore, there are plausible links between neurotransmitters implicated in this disorder and altered functioning of the NVU, though their significance remains to be tested in a clinical context.

## Synthesis

**Figure 1:**
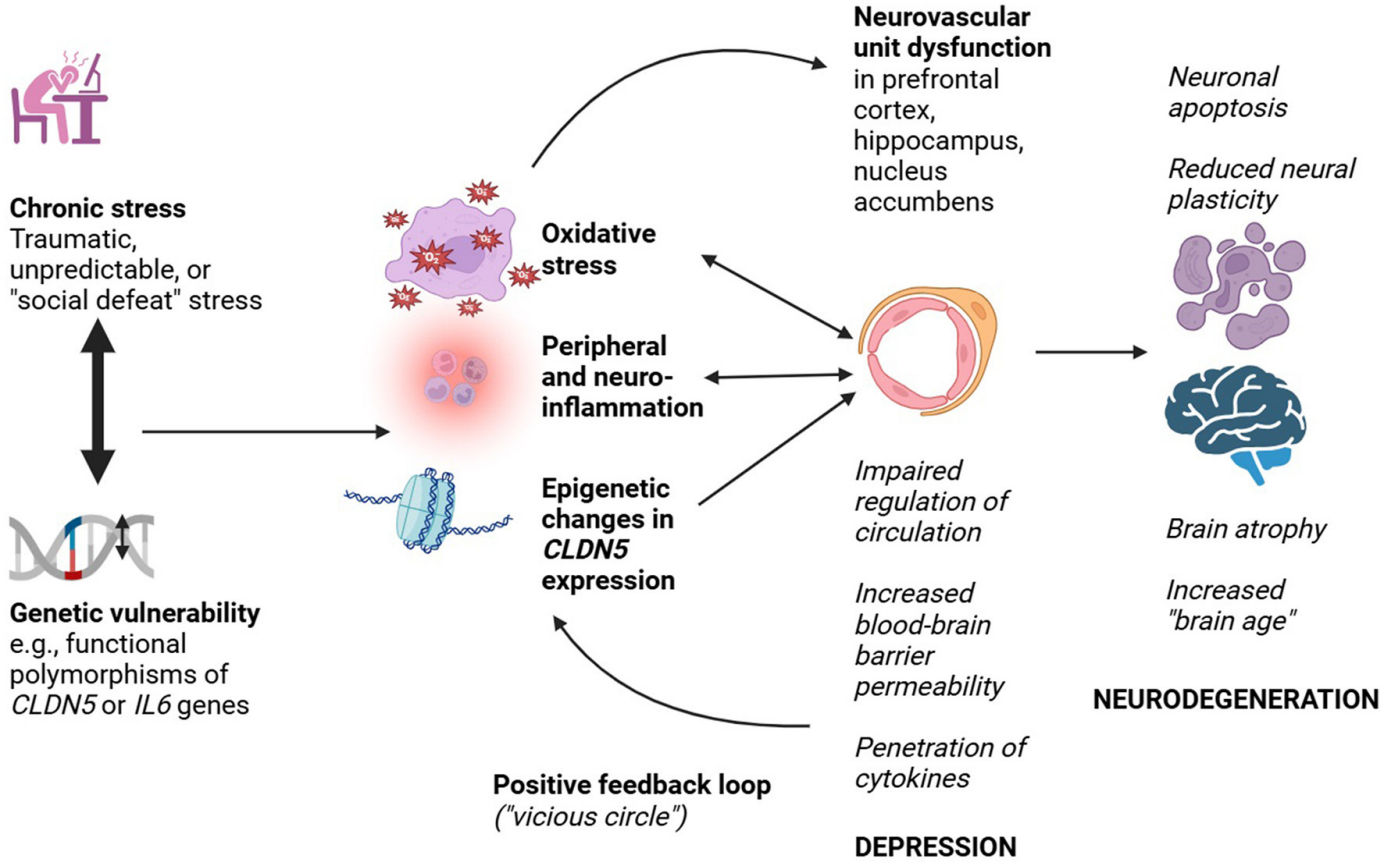
Probable mechanisms linking neurovascular unit dysfunction to depression and neurodegeneration.

A summary of the above findings, and their implications for an NVU-mediated link between depression and neurodegeneration, is presented in Figure 1.

On the one hand, there is no research directly confirming Najjar et al.'s hypothesis of nitric oxide synthase uncoupling being the key link between NVU dysfunction and depression. On the other hand, there is substantial translational and clinical evidence to support a broader version of this hypothesis—namely, that stress-induced NVU dysfunction may be central to the pathophysiology of MDD.

The picture that emerges from this evidence is that stress may impair NVU function in various ways, including epigenetic repression of the *CLDN5* gene and hypothalamic-pituitary-adrenal (HPA) axis leading to increased peripheral inflammation and oxidative stress. This leads to impaired regulation of regional brain circulation, increased permeability of the BBB, the penetration of inflammatory cells and pro-inflammatory cytokines, and a process of NVU “redesign”. Such changes are probably associated with the development of depressive symptoms. Over a longer period of time, increased BBB permeability and impaired vascular regulation—in concert with other risk factors—could increase vulnerability to neurodegeneration through reduced neural plasticity, increased neuronal apoptosis, increased cerebral “aging”, and increased cerebral atrophy. Though it is unlikely to be the sole mechanism involved, dysfunction of the NVU provides a biologically plausible explanation for the association between depression and both Alzheimer's and vascular dementias. A similar explanation can be invoked to explain the apparent link between MDD and subsequent Parkinson's disease. In the latter case, a genetically determined tendency toward the aggregation of alpha-synuclein, which can disrupt NVU/BBB integrity, may act synergistically with the NVU changes caused by stress and depression. This could lead to neuronal loss and neurodegeneration in specific circuits linking the cortex, basal ganglia and brainstem (Hourfar et al., 2023; Wang et al., 2023).

Recent evidence also permits the formulation of two provisional extensions or corollaries to the Najjar et al. hypothesis:

- **Reversibility and irreversibility:** Though NVU dysfunction may be associated with depression in general, this does not necessarily imply that such dysfunction is always progressive or irreversible. In experimental animals, the NVU and BBB changes associated with models of depression have been found to improve after the administration of specific pharmacological agents. These include the antidepressant fluoxetine (Sun et al., 2024), the mood-stabilizing agent lithium (Taler et al., 2021), and the anti-inflammatory drug meloxicam (Pearson-Leary et al., 2017). A tentative association between antidepressant response and a putative marker of NVU disruption has also been demonstrated in patients with MDD (Fornaro et al., 2013). Moreover, the association between MDD and subsequent cerebrovascular disease or neurodegeneration is not fixed. It has been estimated that MDD increases the risk of subsequent PD approximately 2.2-fold (Wang et al., 2018), of AD 2-fold (Hersi et al., 2017), and vascular dementia 2.5-fold (Zhao et al., 2022). Though these figures represent a substantial increase, they also indicate that not all—or even most—patients with MDD will progress to one of these disorders. The most parsimonious explanation for this finding is the first proposed corollary to Najjar et al.'s hypothesis: *initial or milder episodes of MDD may cause less severe and reversible, “functional” alterations of the NVU, which may respond to early and appropriate treatment. On the other hand, with exposure to chronic stress, or after recurrent or chronic MDD, a less reversible “remodeling” of the NVU, with increased oxidative stress, neuroinflammation, neuronal apoptosis and cerebral atrophy, may occur and may increase the risk for subsequent neurodegenerative disorders*. If this corollary is correct, one would expect the duration or severity of depression to correlate with the risk of neurodegeneration. There is some evidence for such a “dose-response” relationship in the case of AD (Hersi et al., 2017). This possibility could be tested by the longitudinal assessment of markers of NVU dysfunction over the course of MDD, and examining the correlations between these markers and the number or severity of major depressive episodes.
- **Specific associations with vascular depression:** The second corollary is that *the relationship between NVU dysfunction, depression and subsequent neurodegeneration would be amplified or accelerated in patients with other “vascular” risk factors, such as diabetes mellitus*. This idea, which has already been proposed by some experts (Duarte-Silva et al., 2021), follows from the results of research in animal models of diabetes mellitus and depression. In these models, stress and hyperglycemia appear to act synergistically to cause disrupted NVU integrity, the production of the excitotoxin quinolinic acid, neuroinflammation, and neuronal apoptosis (Liu et al., 2018; Gliozzi et al., 2024). Similarly, high systolic blood pressure in animals has been found to impair endothelial function and disrupt the NVU, and these changes have been associated with impaired learning and memory (de Montgolfier et al., 2019). This could be linked to the clinical observation that systemic hypertension increases the risk for depression about 2.7-fold (Armstrong et al., 2017), and that both hypertension and depression are risk factors for vascular cognitive impairment and dementia (Sapsford et al., 2022). If this speculation is correct, one would expect to find greater evidence of NVU dysfunction in patients with MDD and comorbid vascular risk factors; this possibility could be tested both cross-sectionally and longitudinally.

## Limitations of the available evidence

Certain precautions are necessary when appraising the evidence linking the NVU to MDD and its long-term neurological outcomes. First, the complete version of Najjar et al.'s hypothesis, involving increased oxidative stress as the driver of NVU dysfunction, has not been formally tested in translational models, and cannot be directly tested with ease in patients with MDD. Second, most of the available research has not yet been independently replicated, and the heterogeneity in models and outcome variables across studies precludes a rigorous synthesis of their findings. Third, human studies have used indirect measures of NVU dysfunction, have been based on small samples, and have used cross-sectional designs that cannot establish causal relationships. Fourth, only a few studies have specifically examined the link between NVU functioning and depression in the context of other vascular risk factors. Fifth, most animal models have relied on the induction of depressive-like behaviors using chronic stress. It is unclear whether the NVU changes in these models correlate more strongly with stress or with depression. Finally, the few clinical studies that have tried to link NVU dysfunction to depression and dementia have yielded inconsistent results, though this may reflect methodological limitations (Hampel et al., 1997; Sinclair et al., 2023). For these reasons, the conclusions drawn above should be considered tentative.

## Conclusions

Alterations in the normal functioning of the NVU are implicated in the pathogenesis of several neuropsychiatric disorders. Recent evidence suggests that NVU dysfunction may be important in determining the onset and course of depression, as well as its possible progression to neurodegenerative disorders. This dysfunction is closely linked to neuroinflammation and oxidative stress, and may involve alterations in tight junction proteins such as claudin-5, neurotransmitters such as glutamate and serotonin, cytokines such as IL-1β and TNF-α, and factors that affect the expression of proteins important for NVU and BBB integrity. These changes may be reversible to a certain extent, providing a possible window for early pharmacological or psychosocial interventions. Future translational research should examine whether dementia-like phenotypes, such as impaired memory, are associated with NVU dysfunction in stress-related animal models of depression. Translational work should also focus on elucidating the relative contributions of distinct cell types to NVU dysfunction in animal models of depression. Human studies with a longitudinal design, examining the correlations between diverse molecular and imaging markers, could provide robust confirmation of NVU dysfunction in depression, both with and without vascular risk factors such as diabetes mellitus, and establish its possible relationship to neurodegeneration. It would be equally informative to examine genetic or environmental risk or protective factors associated with the onset and progression of NVU dysfunction over the course of MDD. Ultimately, such knowledge can inform the development and evaluation of strategies that could both improve treatment outcomes in depression and prevent its progression to certain types of neurodegenerative disorder.

## Author contributions

RR: Conceptualization, Formal analysis, Investigation, Methodology, Supervision, Validation, Visualization, Writing – original draft, Writing – review & editing.
